# Tauroursodeoxycholic Acid Supplementation in In Vitro Culture of Indicine Bovine Embryos: Molecular and Cellular Effects on the In Vitro Cryotolerance

**DOI:** 10.3390/ijms241814060

**Published:** 2023-09-14

**Authors:** Elisa Mariano Pioltine, Camila Bortoliero Costa, Fernanda Fagali Franchi, Priscila Helena dos Santos, Marcelo Fábio Gouveia Nogueira

**Affiliations:** 1 Multi-User Laboratory of Phytomedicines Pharmacology, and Biotechnology (PhitoPharmaTec), Department of Pharmacology, Institute of Biosciences, São Paulo State University (UNESP), Botucatu 18618-000, Brazil; 2 Laboratory of Embryonic Micromanipulation, Department of Biological Sciences, School of Sciences and Languages, São Paulo State University (UNESP), Assis 19806-900, Brazil

**Keywords:** endoplasmic reticulum (ER), ER stress, embryo, in vitro culture, TUDCA, vitrification

## Abstract

During embryo development, the endoplasmic reticulum (ER) acts as an important site for protein biosynthesis; however, in vitro culture (IVC) can negatively affect ER homeostasis. Therefore, the aim of our study was to evaluate the effects of the supplementation of tauroursodeoxycholic acid (TUDCA), an ER stress inhibitor, in the IVC of bovine embryos. Two experiments were carried out: Exp. 1: an evaluation of blastocyst rate, hatching kinetics, and gene expression of hatched embryos after being treated with different concentrations of TUDCA (50, 200, or 1000 μM) in the IVC; Exp. 2: an evaluation of the re-expansion, hatching, and gene expression of hatched embryos previously treated with 200 µM of TUDCA at IVC and submitted to vitrification. There was no increase in the blastocyst and hatched blastocyst rates treated with TUDCA in the IVC. However, embryos submitted to vitrification after treatment with 200 µM of TUDCA underwent an increased hatching rate post-warming together with a down-regulation in the expression of ER stress-related genes and the accumulation of lipids. In conclusion, this work showed that the addition of TUDCA during in vitro culture can improve the cryotolerance of the bovine blastocyst through the putative modulation of ER and oxidative stress.

## 1. Introduction

Disturbances of the endoplasmatic reticulum (ER) homeostasis cause protein folding or misfolding in the ER lumen, a condition called ER stress, which triggers unfolded protein response (UPR) [1]. A paradox of the UPR pathway is that it leads to a response with the simultaneous activation of cell survival and pro-apoptotic pathways. Under those ER stress conditions, the activation of the UPR reduces unfolded protein load through several pro-survival mechanisms, including the expansion of the ER membrane, the selective synthesis of key components of the protein folding and the quality control machinery, and the attenuation of the influx of proteins into the ER [2]. When ER stress is not mitigated and the homeostasis is not restored, the UPR triggers apoptosis. There are three predominant and unique signaling transduction mechanisms among the UPR signaling pathways: inositol-requiring enzyme 1 (IRE1), protein kinase RNA (PKR)-like kinase (PERK), and activating transcription factor 6 (ATF6) [3,4]. The IRE1 endoribonuclease is activated through dimerization and transphosphorylation. This leads to the removal of a 26-nucleotide intron from the premature unmodified form of XBP1 (XBP1-u) gene to produce the unmodified XBP1 (XBP1-s) form [5]. XBP1-s moves to the nucleus and induces UPR-responsive genes. XBP1-s is usually regarded as a reliable marker for the induction of the IRE1 pathway of the UPR, because XBP1 is unmodified exclusively under ER stress conditions [6]. However, if ER stress is excessive or prolonged, the UPR fails, and cellular apoptosis is induced by the activation of CCAAT-enhancer-binding-protein homologous protein (CHOP), Jun N-terminal kinase, and cleaved caspase 3 [7,8].

Early embryonic stages are (one of) the most critical periods of mammalian development [9]. These early stages involve various morphological and biochemical changes related to genomic activity and a complex set of physiological processes, many of which are still unknown [10]. These processes are controlled by several molecular mechanisms and pathways that have a fundamental role in the coordination of homeostatic and metabolic processes [11,12]. Within in vitro systems, disturbances in the embryo’s culture environment after fertilization can have detrimental effects on embryonic gene expression [13], which, in turn, can have serious implications for the normality of the blastocyst’s physiology. However, the exact influence of in vitro culture conditions during each of these critical events/steps is still unknown.

Recent studies in several species have shown that ER stress in the embryo impairs embryo developmental competence [14,15] and that stress relief can improve the embryo quality [15,16,17,18]. In the bovine species, the supplementation of the in vitro culture (IVC) medium with tauroursodeoxycholic acid (TUDCA)—a bile acid that acts as a potential chemical chaperone against ER stress in vitro [19]—has been an alternative to relieve ER stress and improve the developmental competence in embryos cloned by somatic cell nuclear transfer [16] and embryos subjected to high O_2_ tension [20]. In addition, the supplementation of TUDCA at in vitro maturation has been shown to be beneficial in relieving ER stress in the oocyte [21,22,23,24,25].

Nevertheless, little is known about the effects of ER stress on the cryogenic tolerance of embryos generated in vitro [26]. It has been studied that the in vitro-produced embryo (IVPE) has distinct characteristics when compared with its counterpart produced in vivo. These differences between IVPE and in vivo derived from embryos involve morphological [27] and molecular aspects that affect the embryo quality and development [28], decreasing cryo-tolerance and pregnancy rates [29]. Therefore, maintaining cell viability after warming is a prerequisite to achieving high outcomes within cryopreservation protocols. To help bridge this gap between IVPE and in vivo derived from embryos to improve post-cryopreservation results, the ER stress could be an alternative target for a pharmacological approach.

We hypothesized that the supplementation of TUDCA during IVC decreases the endoplasmic reticulum stress of bovine embryos and improves the cryogenic competence of the embryo related to re-expansion and post-warming hatching rates. Thus, the present study aimed (1) to evaluate the developmental competency of bovine embryos after being treated with TUDCA in IVC and (2) to investigate the effects of TUDCA treatment in IVC on the subsequent developmental competency post-warming of the vitrified blastocysts.

## 2. Results

### 2.1. The Effects of TUDCA during IVC of Bovine Embryos

#### 2.1.1. Developmental Competence

**Figure 1:**
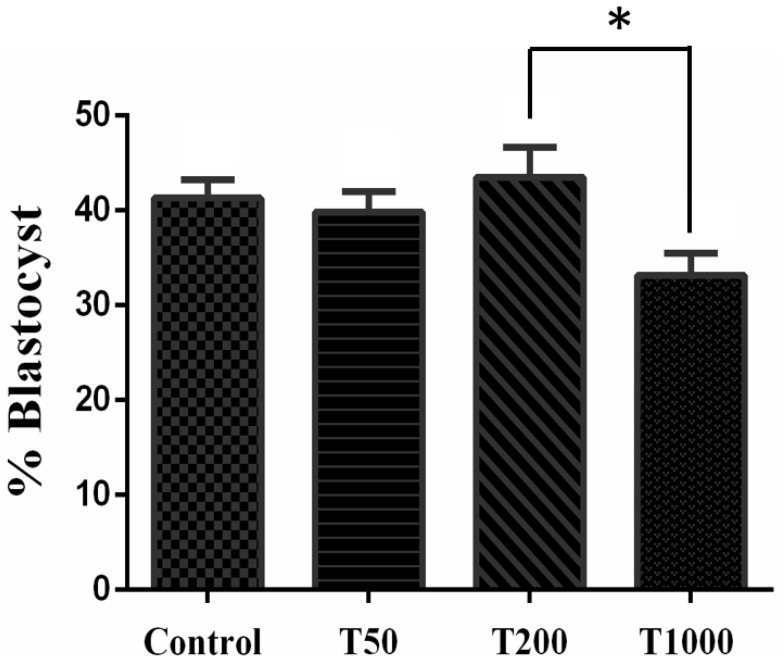
The effect of TUDCA concentrations on the percentage blastocyst. Results were least-squares means ± SEM. * *p* ≤ 0.1. Control = 0 µM of TUDCA; T50 = 50 µM of TUDCA; T200 = 200 µM of TUDCA; T1000 = 1000 µM of TUDCA.

**Figure 2:**
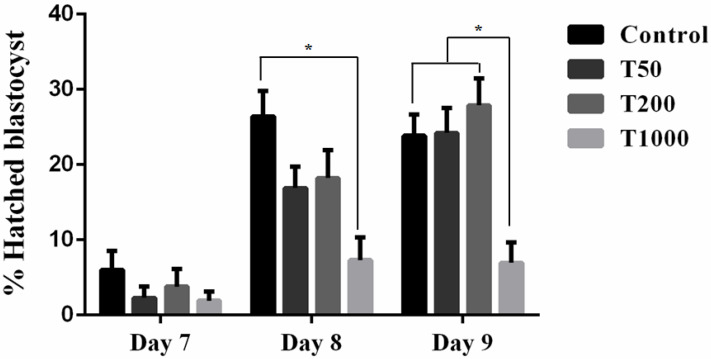
The effect of TUDCA concentrations on the blastocyst hatching kinetics. Results were least-squares means ± SEM. * *p* ≤ 0.1. Control = 0 µM of TUDCA; T50 = 50 µM of TUDCA; T200 = 200 µM of TUDCA; T1000 = 1000 µM of TUDCA.

After 7 days of culture, in the absence and in the presence of TUDCA, there was no difference in the rate of blastocysts among the Control group and the treatments with TUDCA. However, group T200 presented a higher rate of blastocysts when compared with group T1000 (*p* = 0.0268; Figure 1). When evaluating the embryo hatching kinetics, there was no difference between treatments on day 7. However, on days 8 and 9, the T1000 group (7.3% and 6.95%, respectively) was statistically different from the day 8 Control (26.4%) and all the treatments by day 9 (23.8%, 24.2%, and 27.9%, respectively, to Control group, T50, and T200; Figure 2). The results suggested a toxic effect in the group treated with 1000 µM of TUDCA altering the embryonic development kinetics and, subsequently, decreasing its ability to hatch.

#### 2.1.2. Gene Expression

**Figure 3:**
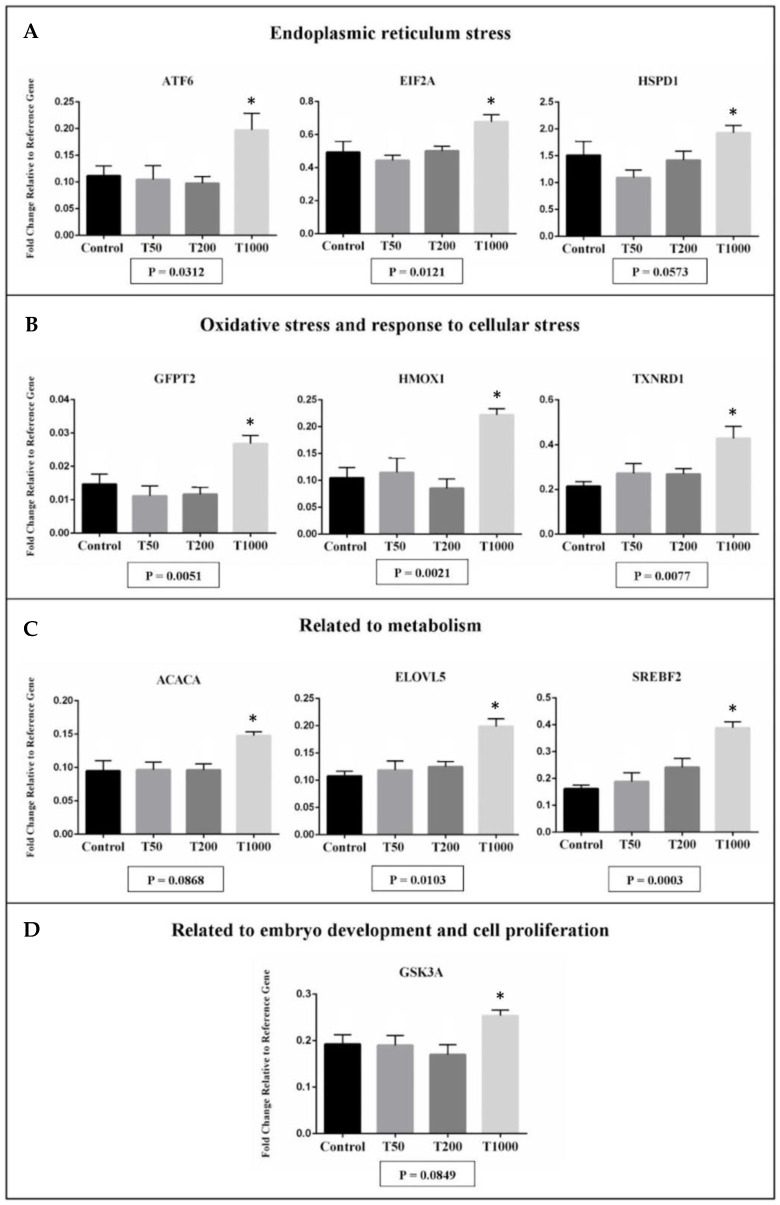
The effect of TUDCA concentrations on mRNA abundance of candidate expressed genes in the embryo according to the following functional categories: endoplasmic reticulum stress (**A**), oxidative stress and response to cellular stress (**B**), related to metabolism (**C**), and related to the embryo development and cell proliferation (**D**). Results were least-squares means ± SEM. * *p* ≤ 0.1. Control = 0 µM of TUDCA; T50 = 50 µM of TUDCA; T200 = 200 µM of TUDCA; T1000 = 1000 µM of TUDCA.

The transcript abundance from ten genes was significantly affected in hatched blastocyst after TUDCA treatment, with all genes upregulated in the T1000 group in comparison with the other groups (Figure 3). Of the candidate expressed genes, there were transcripts related to endoplasmic reticulum stress (ATF6, EIF2A, and HSPD1), oxidative stress and response to cellular stress (GFPT2, HMOX1, and TXNRD1), metabolism (ACACA, ELOVL5, and SREBF2), and the embryo development and cell proliferation (GSK3A; Figure 3). Corroborating with the analysis of the developmental competence, the T1000 group appeared to be toxic to the embryo, increasing the abundance of transcripts related to ER stress and oxidative stress.

### 2.2. The Effects of TUDCA during IVC on Post-Warmed Vitrified Blastocysts

#### 2.2.1. Developmental Competence

There was no difference in the re-expansion rate evaluated after 12, 24, and 48 h of warming up (*p* > 0.1; Table 1). Nevertheless, a larger number of hatched blastocysts were observed from the TUDCA treatment in the assessment post-warming (Table 1). After 24 and 48 h post-warming, there was a higher number of hatched blastocysts in the embryos cultivated with TUDCA compared with the Control (*p* = 0.09 and 0.0423, respectively; Table 1). The results suggested a beneficial effect of treatment with TUDCA by increasing the hatching rates of embryos subjected to vitrification.

#### 2.2.2. Gene Expression

**Figure 4:**
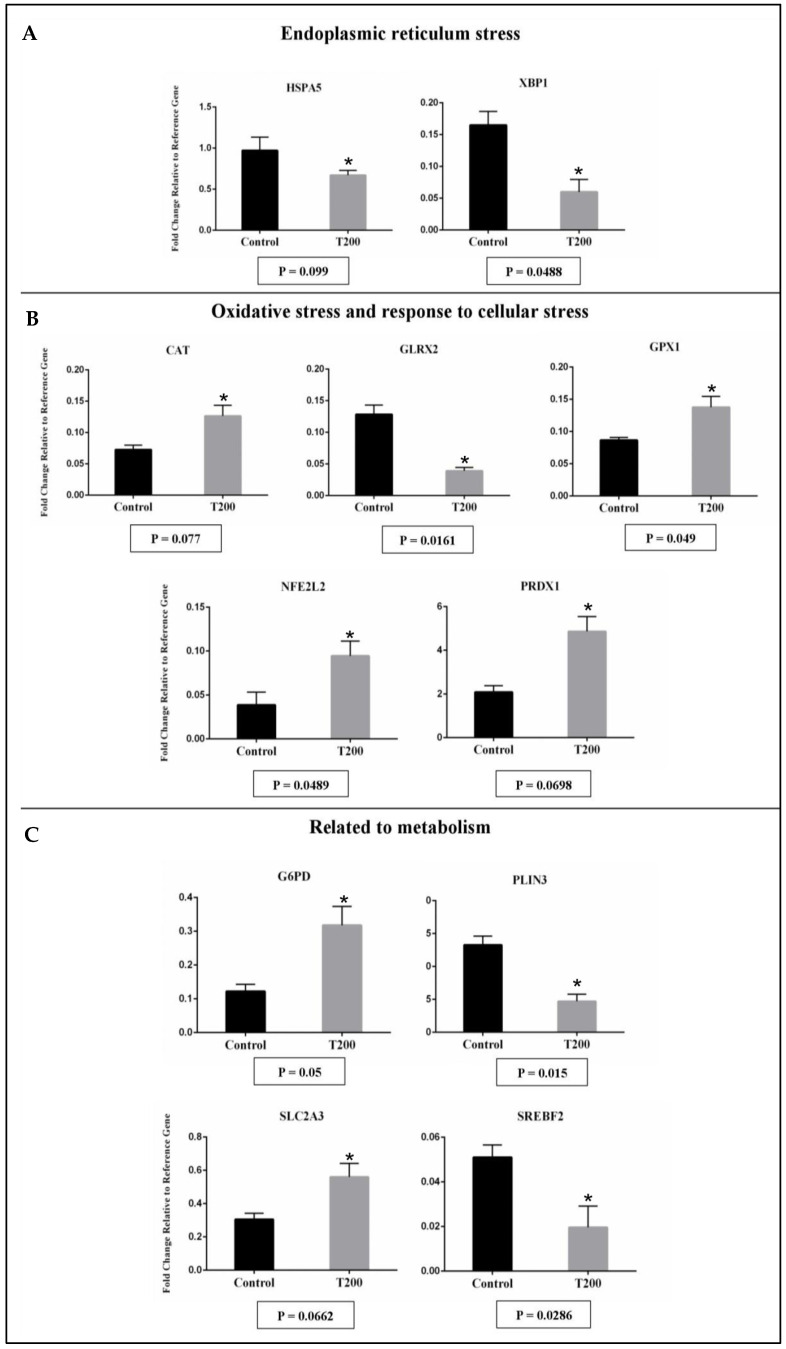
The effect of TUDCA concentrations on mRNA abundance of candidate expressed genes in the embryo according to the following functional categories: endoplasmic reticulum stress (**A**), oxidative stress and response to cellular stress (**B**), and related to metabolism (**C**). Results were least-squares means ± SEM. The effect of treatment was * *p* ≤ 0.1 for all genes in the figure. Control = 0 µM; T200 = 200 µM.

The transcript abundance from 11 genes was affected in hatched blastocyst after vitrification and post-warming. When compared with the Control group, in the T200 group, five genes were down-regulated, and six genes were up-regulated (Figure 4). Down-regulation was observed for genes related to endoplasmic reticulum stress (HSPA5 and XBP1), oxidative stress and response to cellular stress (GLRX2), and some markers for metabolism (PLIN3 and SREBF2; Figure 4). Up-regulation was observed for genes related to oxidative stress and response to cellular stress (CAT, GPX1, NFE2L2, and PRDX1) and metabolism (G6PD and SLC2A3; Figure 4). The treatment with TUDCA could have relieved the ER stress and modulated the lipid metabolism of the hatched embryo, but direct evaluations of these pathways were not performed. String analysis of those genes that are statistically different reveals a correlation between genes related to endoplasmic reticulum stress and genes related to oxidative stress and response to cellular stress (Supplementary Material Figure S1).

## 3. Discussion

Developing embryos may be subjected to several sources of exogenous stress in an in vitro culture system [15,30,31,32]. These include oscillating temperature, DNA damage or DNA damaging agents, osmotic stress, and the availability of organic osmolytes, oxygen and oxidative stress, hyperglycemia and carbon substrate availability, hyperlipidemia and oxidized lipids, calcium ionophores, cytokines, amino acid deprivation, insulin signaling, and serum components [15,30,31,32]. Cold stress associated with cryopreservation affects embryo development and gene regulation [27,29,33]. These adverse factors negatively impact ER functions and protein synthesis and folding, resulting in the activation of ER stress and the UPR signaling pathways in in vitro-produced embryos [14,15,16,17]. Furthermore, there is evidence that IVPEs are more sensitive to cryopreservation than in vivo derived from embryos [34,35] and that this reduced cryotolerance may be associated with the high lipid content present in the cytoplasm as well as the lipid profile of the cell membrane of these embryos [28,29,34]. Although TUDCA has been demonstrated to exert efficient cytoprotective activity in relieving ER stress [17,18,20,26], recent studies reported on its new potential and molecular modes of action as a weight-reducing agent, modulating lipid metabolism through or independently of ER modulation [36,37]. In the current study, we demonstrated that 200 µM of TUDCA during IVC enhanced the cryotolerance of bovine embryos through the putative modulation of ER and oxidative stress. At 24 and 48 h post-warming, embryos treated with TUDCA during IVC had an increase in hatching rate when compared with the Control group.

Unlike our results, it was reported in cattle that 10 µM TUDCA was able to improve the cryotolerance of embryos after vitrification, increasing hatching rates and decreasing the number of apoptotic cells in the embryo 48 h post-warming [26]. In this way, the action of TUDCA seems to be highly dependent on the complex combination of variables such as the species, breed, the used concentration, and the in vitro culture conditions.

When we evaluated the 96 markers of transcript abundance in the hatched embryos post-warming, TUDCA treatment induced the decreasing of the mRNA abundance related to ER stress and lipid metabolism pathways. An increase in the mRNA abundance related to antioxidant activity was also observed in embryos in the T200 group. After warming post-vitrification, hatched blastocysts treated with TUDCA showed less mRNA abundance for HSPA5 and XBP1. In several species, the increase of XBP1-s expression is widely used as a molecular marker of ER stress in vivo and in vitro [38,39]. The increasing mRNA abundance for XBP1-s and HSPA5 was associated with the low competence of embryonic development in several species [18,20,35]. In addition, post-warming vitrified embryos treated with TUDCA showed a change in the expression of antioxidants, with a high mRNA abundance for CAT, GPX1, NFE2L2, and PRDX1, and less abundance for GLRX2. NFE2L2 is a promising target against oxidative stress, responsible for inducing the expression of several endogenous cytoprotective enzymes [40,41]. An in vitro study with the human neuroblastoma SH-SY5Y cell line observed that TUDCA prevented oxidative stress through the highest expression of NRF2, DJ-1, and antioxidant enzymes heme oxygenase-1 (HO-1) and glutathione peroxidase (GPx) [42], corroborating with our results. The previous study has shown that embryos produced in vivo and cryopreserved undergo greater oxidative stress when compared with embryos that have not been subjected to cryopreservation [33]. In the conditions of this paper, the treatment with TUDCA could have prevented high levels of oxidative stress in vitrified embryos cultured after warming. Collectively, these data could partially explain the higher cell competence to hatch observed with the TUDCA treatment, since a mitigated stress (ER and oxidative) improves the cellular activity.

The hatched embryos also modulated markers related to the metabolism. While the mRNA abundance for PLIN3 and SREBF2 decreased, the abundance for G6PD and SLC2A3 increased with the TUDCA treatment.

The reduced post-warming cell viability is also associated with the abnormal amount and/or the type of lipids in the blastomeres that contributes to the occurrence of cryogenic fractures during the freezing process [28,34]. The cell membrane fluidity is related to the lipid profile and the capacity to support cryo-injuries during the cryopreservation process [34]. The IVPEs have a different lipid profile from their in vivo-derived counterparts [34,35] and could possess a cryotolerance enhanced by changes in the lipid metabolism (e.g., TUDCA treatment). Although the evaluation of the lipid content was not assessed in this work, we cannot rule out that TUDCA treatment may have modulated the lipid content of the hatched embryo (down-regulated mRNA abundance of PLIN3 and SREBF2 in T200 group).

In addition, G6PD (Glucose-6-phosphate dehydrogenase) and SLC2A3 (Solute carrier family 2, facilitated glucose transporter member 3) mRNAs were up-regulated in the embryo treated with TUDCA. It was reported that those genes were essential for pre-implantation embryonic development [43,44,45]. Partially, this finding added data to explain how the TUDCA treatment could improve the post-warming hatching rate of vitrified embryos. Since the mammalian hatching process has involved a coordinated trophectoderm activity and is concomitant with the beginning of hypoblast appearance in bovine species [46], the up-regulation of G6PD and SLC2A3 genes suggest a positive marker after TUDCA treatment.

TUDCA supplementation in IVC was associated with improved embryonic developmental rates in mice [17,23], pigs [18,21], and cows [20,26]. In our experiment, we did not find a significant increase in the rates of blastocyst formation with TUDCA treatments. Similarly, in conditions of low O_2_ tension (5%), the supplementation of 50 µM of TUDCA in the IVC of bovine embryos did not modulate embryo competence [20]. However, embryos that were submitted to high tension O_2_ (20%) in the IVC showed an increased blastocyst rate in cattle and pigs with the supplementation of, respectively, 50 and 200 µM of TUDCA [18,20]. Once again, the potential beneficial effect of TUDCA supplementation seems to be linked with the culture conditions. When the stringent condition of the embryo culture is used (e.g., high oxygen tension as source to generate an increase in the reactive oxygen species), the effect of TUDCA to alleviate ER stress was observed [18,20], but this was not the case in our study. Corroborating with our result, some other recent studies have shown that the increase in reactive oxygen species (ROS), due to O_2_ tension, is closely related to the increase in ER stress in embryos [20]. In addition, unlike the purpose of our study, TUDCA proved to be beneficial for the development of in vitro-produced embryos in conditions where ER stress was chemically or physically actively induced (e.g., using tunicamycin or heat stress, respectively) [18,20,26,47,48,49].

Complementing these results, when we evaluated the hatching kinetics of embryos treated with TUDCA, a significant reduction in the hatched embryos rate was observed with the T1000 group when compared with the other groups (with or without the addition of TUDCA). Contrary to that reported in mice—which had a positive effect on embryonic development and the newborn rate was described with the addition of 1000 µM of TUDCA in the culture [23]—the higher concentration of TUDCA used in our study proved to be toxic to the bovine embryo and impaired its development. Furthermore, the factor already mentioned (O_2_ tension linked to the TUDCA effect) seems to suggest that the species (mouse or cattle) also plays a role on the upper threshold of the beneficial effect of TUDCA (i.e., when the threshold is exceeded, and the toxic effect is observed).

In the analysis of the mRNA abundance involved in ER stress, oxidative stress, metabolism, and embryonic quality, the negative effect on the hatching rate of the T1000 group was reinforced.

In the case of misfolded proteins in the ER lumen, molecular chaperones (HSPD1 and HSPA5) are activated in order to correct this misfolding and maintain homeostasis in the ER [50,51]. For instance, in cases of ER stress, HSPA5 dissociates from PERK, ATF6, and IRE1 receptors, activating the UPR pathway [3,4,50]. Activated PERK can recognize and phosphorylate eIF2α, which in turn positively regulates the translation of ATF4, an important inducer of CHOP, GADD34, ATF-3, and genes involved in apoptosis [52]. In the T1000 group, there was an increase in the transcript abundance for HSPD1, ATF6, and EIF2A in the hatched embryos. This suggests that the higher concentration of TUDCA of this study paradoxically induced ER stress in the blastocysts. Also, an increase in the gene’s expression involved with oxidative stress (e.g., GFPT2, HMOX1, and TXNRD1) was observed and this reinforced the close relationship between ER stress and oxidative stress, where ROS functioned as a mediator of these two events [53,54]. Oxidative stress in embryos could lead to DNA damage [30] and inhibit preimplantation development [55]. For transcripts involved in lipid metabolism (SREBF2, ELOVL5, and ACACA), there was an up-regulation in embryos of the T1000 group. Crosstalk between ER stress and lipid metabolism was well established [56,57,58,59]. Several reports indicated that the pathways that regulate UPR also induce lipid accumulation in the cell. For instance, the ATF6α pathway plays a role in lipid accumulation interacting with the nuclear form of SREBP-2 [58,59]. In the literature, the lipid accumulation in embryos is associated with lower rates of embryonic survival after cryopreservation and deviations in the relative abundance of transcripts of important genes for embryonic development [28,34]. Additionally, in the corroborating results, the T1000 group has been shown to increase the transcript abundance for GSK3A (related to embryo development and cell proliferation). GSK3A is a negative regulator in the hormonal control of glucose homeostasis, cell division, proliferation, motility, and survival. In other publications, the highest expression of GSK3A in the embryo is associated with low embryo competence [60].

Unlike the T1000 group, the T50 and T200 groups did not significantly affect gene expression in hatched blastocysts when compared with the Control, corroborating the results of embryonic development. Although without molecular and cellular evidence of any beneficial effect of TUDCA (T50 and T200 groups), there was no assessment on the pregnancy rate of those embryos (fresh transfer) or the effect of a high oxygen culture system.

## 4. Materials and Methods

### 4.1. In Vitro Production

Bovine ovaries (mainly Bos t. indicus and its crossbreeds) were obtained from a commercial abattoir located at Assis (São Paulo, Brazil) during the months of August and September. They were transported to the laboratory in sterile saline (0.9% NaCl) at 37 °C for 30 min at maximum. Cumulus oocyte complexes (COCs) were collected through aspiration of follicles 3–8 mm in diameter [61]. After sedimentation, COCs were recovered and selected using a stereomicroscope. Only the COCs with a homogeneous cytoplasm and a compact multilayer of cumulus cells were used (grades 1 and 2) [62]. COCs were washed and transferred to 500 µL drops of maturation medium (10 µL/COCs) in 4-well dishes (Nunc, Roskilde, Denmark), which consisted of TCM199 containing Earle salts supplemented with 0.1 IU/mL rhFSH (Gonal-f, Merck Serono, Rockland, MA, USA), 0.22 mg/mL sodium pyruvate, 75 µg/mL amikacin, and 4 mg/mL BSA. Drops were incubated at 38.5 °C in humidified air with 5% CO_2_ for 24 h.

Following in vitro maturation (IVM), groups of 25 COCs were transferred to 90 µL drops of Tyrode Albumin Lactate Pyruvate (TALP) supplemented with fatty-acid-free BSA (6 mg/mL), pyruvate (0.22 mg/mL), amikacin (75 µg/mL), heparin (30 µg/mL), and PHE (20 µM penicillamine, 10 µM hypo taurine, and 1 µM epinephrine). Oocytes were subjected to in vitro fertilization (IVF) step with frozen–thawed semen from a single sample of a Nellore breed bull (named “Ópio”). Spermatozoa were selected using the Select SPERM (Botupharma Animal Biotechnology, Botucatu, São Paulo, Brazil) method, and the concentration was adjusted to 1 × 10^6^ spermatozoa/mL. Oocytes and spermatozoa were co-incubated under the same conditions as during IVM, and the day of insemination was designated as Day 0. At 18–20 h post-insemination, presumptive zygotes were denuded from cumulus cells and transferred to 500 µL drops of SOF medium (synthetic oviduct fluid; 10 µL/zygotes) in 4-well dishes, supplemented with pyruvate (0.22 µg/mL), amikacin (75 µg/mL), 2.5% *v*/*v* of FCS and BSA (5 mg/mL), and concentrations of TUDCA according to the experimental design described below. All experiments had a Control group (i.e., culture medium without the addition of TUDCA). Subsequently, they were cultivated in physiological oxygen tension (5%) in small, sealed plastic bags with a gas mixture of 5% O_2_, 5% CO_2_, and 90% N_2_ [63], and high humidity in an incubator at 38.5 °C. The culture was maintained to all experiments, for 9 days after insemination to reach the hatching stage of the embryos.

### 4.2. Chemical Treatment

Tauroursodeoxycholic acid sodium salt (TUDCA; Selleckchem, Houston, TX, USA) was dissolved in sterile, distilled water to make a 100 mM stock solution (stored at −80 °C). This stock solution was diluted into culture media to make 50 µM, 200 µM, and 1000 µM solutions of TUDCA at experiment 1 (respectively, groups T50, T200, and T1000) [21,23,64]. Experiment 2 (vitrification) was performed only with the T200 group.

### 4.3. Target-Transcripts Relative Quantitation: RT-qPCR

#### 4.3.1. RNA Isolation and Reverse Transcription

The total RNA from hatched blastocysts was extracted with the PicoPure RNA Isolation kit (Life Technologies, Foster City, CA, USA) following the manufacturer’s protocol. Extracted RNA was stored at −80 °C until further analysis using qPCR. RNA concentration was quantified using a spectrophotometer (Nanodrop, ThermoFischer Scientific, MA, USA).

We used 3 to 5 replicates per experimental group and for each sample (i.e., the replicate), a pool of 3 hatched blastocysts to reverse transcription. The cDNA synthesis was performed using High-Capacity Reverse Transcription kit (Applied Biosystems, Foster City, CA, USA), following manufacturer’s instructions. DNAse treatment was performed in all samples before reverse transcription as manufactures’ instructions.

#### 4.3.2. Preamplification and Quantitative Polymerase Chain Reaction

Gene expression analysis of blastocysts was performed independently, using Applied Biosystems™ TaqMan^®^ Assays, specific for Bos t. taurus. A total of 80 candidate genes was analyzed (Supplementary Material Table S1). Prior to qPCR thermal cycling, each sample was submitted to a sequence-specific preamplification process as follows: 1.25 µL assay mix (TaqMan^®^ Assay was pooled to a final concentration of 0.2× for each of the 96 assays), 2.5 µL TaqMan PreAmp Master Mix (Applied Biosystems, #4391128), and 1.25 µL cDNA (5 ng/µL). The reactions were activated at 95 °C for 10 min followed by denaturing at 95 °C for 15 s, annealing and amplification at 60 °C for 4 min for 14 cycles. These pre-amplified products were diluted 5-fold prior to RT-qPCR analysis.

Assays and pre-amplified samples were transferred to an integrated fluidic circuits (IFC) plate. For gene expression analysis, the sample solution prepared consisted of 2.25 µL cDNA (pre-amplified products), 2.5 µL of TaqMan Universal PCR Master Mix (2×, Applied Biosystems), and 0.25 µL of 20× GE Sample Loading Reagent (Fluidigm, South San Francisco, CA, USA), and the assay solution was as follows: 2.5 µL of 20× TaqMan Gene Expression Assay (Applied Biosystems) and 2.5 µL of 2× Assay Loading Reagent (Fluidigm). The 96.96 Dynamic Array™ Integrated Fluidic Circuits (Fluidigm) chip was used for data collection. After priming, the chip was loaded with 5 µL of each assay solution and 5 µL of each sample solution and was loaded into an automated controller that prepares the nanoliter reactions.

The qPCR thermal cycling was performed in the Biomark HD System (Fluidigm) using the protocol TaqMan GE 96 × 96 Standard, which consisted of one stage of Thermal Mix (50 °C for 2 min, 70 °C for 20 min, and 25 °C for 10 min) followed by a Hot Start stage (50 °C for 2 min and 95 °C for 10 min), followed by 40 cycles of denaturation (95 °C for 15 s), primer annealing, and extension (60 °C for 60 s).

### 4.4. Vitrification of Embryos

#### 4.4.1. Embryo Freezing

All media used for vitrification and for warming were from Vitrogen Ltd. (Cravinhos, São Paulo, Brazil). The Vitrific^®^ device was purchased from WTA Ltd. (Cravinhos, São Paulo, Brazil).

Quality grades 1 and 2 [62] expanded blastocysts were collected on days 7 and 8 of culture (D7 and D8; *n* = 205). Blastocysts were removed from the culture medium (Control group and T200) and washed three times in holding medium (washing medium at 37 °C). After this, a vitrification solution 1 (V1) was used at room temperature, in which the embryos were submitted to two sequential baths for 8 min each. Subsequently, the embryos were transferred to a second solution (vitrification solution 2; V2). In this solution, they remained for just 40 s and then 3 to 5 structures were allocated to the Vitrific^®^ device with the aid of a glass micropipette (approximate inner diameter of 150 μm) to ensure the loading with the minimum possible of the medium. Immediately after that, the structures were deposited on the device and the excess solution was removed, leaving only a thin layer of medium on the structures. Afterwards, the Vitrific^®^ device was plunged directly into the liquid nitrogen (N_2_) in a vertical position. Finally, the protective cap was placed with the device still submerged in N_2_. The blastocysts remained vitrified for an average of 12 to 24 h.

#### 4.4.2. Warming and Culture of Cryopreserved Embryos

For warming, the Vitrific^®^ device was removed from the N_2_ with the aid of tweezers. The device’s cap was removed and, immediately after, its tip containing the structures was submerged into the warmed solution 1 (D1) at 37° C for 1 min. Subsequently, the embryos were washed in two drops of warmed solution 2 (D2) and two drops of warmed solution 3 (D3) at room temperature, with an average time of 6 min for all steps. After that, the embryos were washed in 5 drops of culture medium. Then, the embryos were transferred to 4-well dishes with 500 µL of culture medium (maximum of 50 structures per well) and they were incubated in 38 °C and 5% O_2_ tension in humidified air (IVC conditions). In the end, embryos were evaluated for re-expansion and hatching rate at 12, 24 and 48 h post-warming [26,34].

### 4.5. Experimental Design

#### 4.5.1. Experiment 1: The Effects of TUDCA during IVC on Developmental Competence and Gene Expression of Embryos

To investigate the effect of TUDCA on embryo development and quality, blastocyst and hatched blastocyst rates were analyzed, respectively, on day 7 and days 8–9 (Day 0 being the day of insemination). Hatched blastocysts were collected on days 8–9 for gene expression analysis. After RNA extraction and cDNA reverse transcription, the Biomark HD platform was used to relatively quantify the mRNA abundance of markers of interest. This experiment was replicated five times using 250 presumptive zygotes/treatment.

#### 4.5.2. Experiment 2: The Effects of TUDCA during IVC on Developmental Competence and Gene Expression of Post-Warmed Vitrified Blastocysts

Following the treatment with TUDCA in the culture, expanded blastocysts were collected on days 8–9 and subjected to the technique of vitrification. After 12–24 h, the cryopreserved blastocysts were warmed and cultured at low tension of O_2_. Embryo re-expansion and hatching rates were analyzed at 12, 24, and 48 h post-warming. Hatched embryos were collected 24 and 48 h post-warming for the analysis of gene expression. Later, RNA extraction and cDNA reverse transcription, the Biomark HD platform was used to relatively quantify the mRNA abundance of markers of interest. This experiment was replicated five times using 88 to 117 vitrified embryos/treatment.

### 4.6. Statistical Analysis

To the embryonic development (experiments 1 and 2), the blastocysts and hatched blastocysts rates were arcsines transformed and subjected to analysis of variance (ANOVA), and the means were compared using the post hoc Tukey test. The normality was assessed with the Shapiro–Wilk test and Bartlett’s test. The results were presented as the mean ± standard error of the mean (SEM). For quantitative PCR data, we calculated the ΔCq values relative to the geometric mean of the best reference genes—i.e., B2M, HPRT1, and PPIA (experiment 1) and ACTB, HPRT1, and PPIA (experiment 2)—among the 96-gene set. Fold-changes (Supplementary Materials Tables S2 and S3) were calculated as 2^−ΔCq^. All analyses were performed using JMP software (version number 15, SAS Institute, Cary, NC, USA). Statistical significance was determined based on a *p*-value ≤ 0.1, the significance being considered a trend (between 0.051 and 0.1), moderate (between 0.010 and 0.050), or strong (less than 0.01).

## 5. Conclusions

The present study showed that the addition of TUDCA during in vitro culture can improve the cryotolerance of the bovine blastocyst through the putative modulation of ER and oxidative stress. However, in the culture conditions of this study, there was no observed effect on embryo development with the treatment of 50, 200, and 1000 µM of TUDCA. Moreover, the highest concentration (1000 µM) proved to be detrimental to the development and kinetics of the embryo.

## Figures and Tables

**Table 1:** Re-expansion and hatching rate of embryos after vitrification on Days 7 and 8 (only expanded blastocysts) from groups Control or T200 (200 µM of TUDCA). Total of five replicates (mean ± standard error mean).

Treatment	Vitrified Embryos	Re-Exp. Rate	Re-Exp. Rate	Re-Exp. Rate	Hatch. Rate	Hatch. Rate	Hatch. Rate
	(D7/D8)	12 h (%)	24 h (%)	48 h (%)	12 h (%)	24 h (%)	48 h (%)
Control	88	55.54 ± 5.62	67.19 ± 4.81	67.19 ± 4.81	3.23 ± 1.33	11.71 ± 3.28 ^b^	26.67 ± 4.72 ^b^
T200	117	61.88 ± 3.63	68.22 ± 5.16	79.00 ± 4.51	7.61 ± 2.44	20.50 ± 3.15 ^a^	45.87 ± 4.62 ^a^
*p*-value	-	0.4254	0.8885	0.1112	0.1552	0.09	0.0423

Abbreviations: Re-exp.: re-expansion; Hatch.: hatching. Different letters represent a trend or a significant difference (*p* ≤ 0.1).

## Data Availability

The original data of this present study are available from the corresponding authors.

## Supplementary Materials

The supporting information can be downloaded at: https://www.mdpi.com/article/10.3390/ijms241814060/s1.
