# Supplementary material for: Tauroursodeoxycholic Acid Supplementation in In Vitro Culture of Indicine Bovine Embryos: Molecular and Cellular Effects on the In Vitro Cryotolerance
Source: Int J Mol Sci. 2023 Sep 14;24(18):14060. doi: 10.3390/ijms241814060 (PMC10531190; doi:10.3390/ijms241814060)
Supplement: Supplementary file 1 [file ijms-24-14060-s001.zip › Supplementary data Figure S1 and Table S1.pdf]

Supplementary Materials:

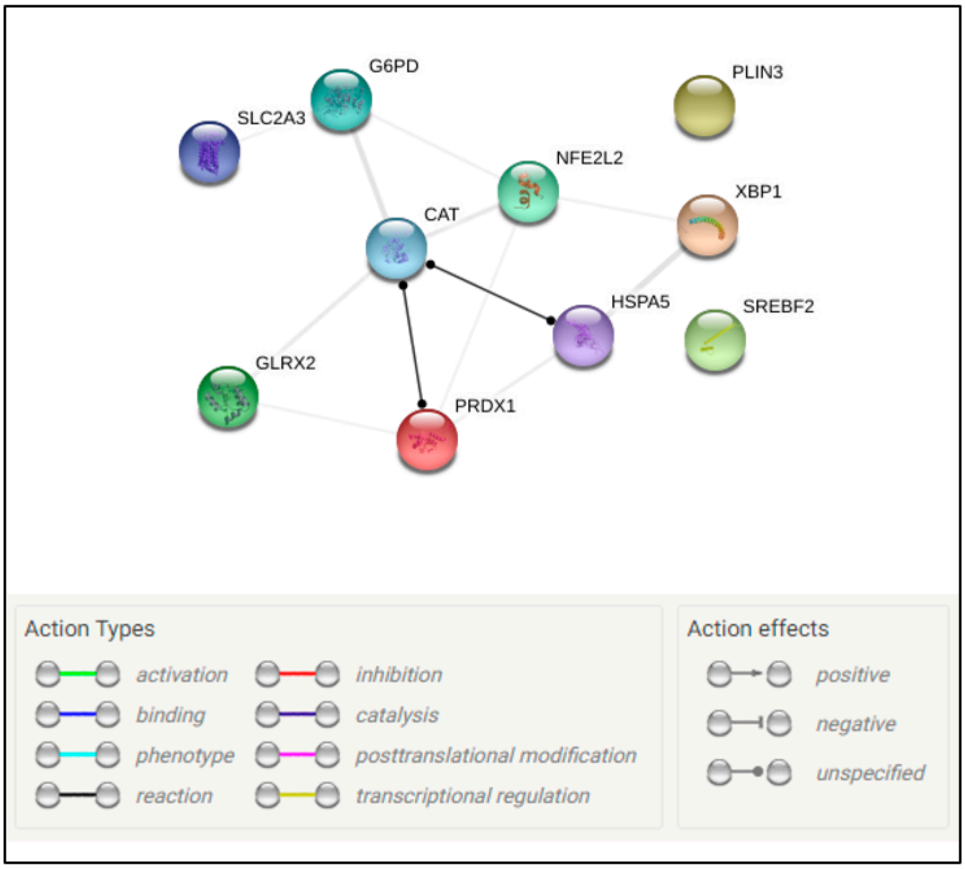

Figure S1. STRING network of protein-protein interactions between genes with trend or significant difference (P≤0.1) of mRNA abundance among groups. Different line colors represent the relationship between the proteins translated from analyzed genes, as described on the legend.

Table S1: Gene symbol, functions and primers assay ID used for microfluidic expression analyses (Biomark HD System - Fluidigm).

Gene Symbol	**Function**	**Assay ID***
PPIA	Reference gene	PA5-16914
GAPDH	Reference gene	Bt03210912_g1
ACTB	Reference gene	Bt03224617_g1

RPL30	Reference gene	Bt03226330_g1
B2M	Reference gene	Bt03251628_m1
HMBS	Reference gene	Bt03234763_m1
HPRT1	Reference gene	Bt03225311_g1
RLP15	Reference gene	Bt03288449_g1
NFE2L2	Oxidative stress and response to cellular stress	Bt03817661_m1
KEAP1	Oxidative stress and response to cellular stress	Bt03228713_m1
CAT	Oxidative stress and response to cellular stress	Bt03215423_g1
SOD1	Oxidative stress and response to cellular stress	Bt03215423_g1
SOD2	Oxidative stress and response to cellular stress	Bt03244551_m1
GPX1	Oxidative stress and response to cellular stress	Bt03259217_g1
GPX4	Oxidative stress and response to cellular stress	Bt03259611_m1
PRDX1	Oxidative stress and response to cellular stress	Bt03223684_m1
PRDX3	Oxidative stress and response to cellular stress	Bt03214402_m1
ARO(CYP19A1)	Oxidative stress and response to cellular stress	Bt03213774_m1
GFPT2	Oxidative stress and response to cellular stress	Bt03250351_m1
GLRX2	Oxidative stress and response to cellular stress	Bt03229700_m1
FOX3	Oxidative stress and response to cellular stress	Bt03649334_s1
TXNRD1	Oxidative stress and response to cellular stress	Bt03215471_m1
VNN1	Oxidative stress and response to cellular stress	Bt03220248_m1
H1F1A	Oxidative stress and response to cellular stress	Bt03259341_m1
HMOX1	Oxidative stress and response to cellular stress	Bt03218624_m1
EIF2A	Endoplasmic reticulum stress	Bt03274460_m1
HSPA5	Endoplasmic reticulum stress	Bt03244880_m1
HSPD1	Endoplasmic reticulum stress	Bt04301470_g1
HSPA1A	Endoplasmic reticulum stress	Bt03292670_g1
ATF4	Endoplasmic reticulum stress	Bt03221057_m1
ATF6	Endoplasmic reticulum stress	Bt03287802_s1
XBP1	Endoplasmic reticulum stress	Bt03227621_g1
DDIT3	Endoplasmic reticulum stress	Bt03251320_g1
DNMT1	DNA methylation	Bt03224737_m1

DNMT3A	DNA methylation	Bt01027164_m1
DNMT3B	DNA methylation	Bt03259810_m1
HP1	DNA methylation	Bt03246076_m1
PAF1	DNA methylation	Bt03239371_g1
NANOG	Related to pluripotency and cell differentiation	Bt03220541_m1
POU5F1(OCT4)	Related to pluripotency and cell differentiation	Bt03223846_g1
HAND1	Related to pluripotency and cell differentiation	Bt04318733_g1
REST	Related to pluripotency and cell differentiation	Bt03278318_s1
IGFBP2	Related to embryo development and cell proliferation	Bt01040719_m1
IGFBP4	Related to embryo development and cell proliferation	Bt03259500_m1
SOX2	Related to embryo development and cell proliferation	Bt03278318_s1
IFITM3	Related to embryo development and cell proliferation	Bt03292973_g1
GSK3A	Related to embryo development and cell proliferation	Bt03273698_g1
KRT8	Related to embryo development and cell proliferation	Bt03225178_g1
LUM	Related to embryo development and cell proliferation	Bt03211920_m1
MTIF3	Related to embryo development and cell proliferation	Bt03231844_m1
TNF	Related to embryo development and cell proliferation	Bt03259156_m1
S100A10	Related to embryo development and cell proliferation	Bt03215645_m1
S100A14	Related to embryo development and cell proliferation	Bt03230771_g1
GATM	Related to embryo development and cell proliferation	Bt03237896_m1
BAX	Related to apoptosis	Bt03211777_g1
CASP9	Related to apoptosis	Bt04282453_m1
CASP3	Related to apoptosis	Bt03250954_g1
BID	Related to apoptosis	Bt03241255_m1
CD40	Related to apoptosis	Bt03817804_g1
1L-1b	Related to apoptosis	Bt03212740_m1
NFKB2	Related to apoptosis	Bt03272789_g1
SREBF1	Related to metabolism	Bt03276370_m1
SREBF2	Related to metabolism	Bt04283467_m1
ACACA	Related to metabolism	Bt03213360_m1
ACSL3	Related to metabolism	Bt04282138_m1

ELOVL3	Related to metabolism	Bt00907566_m1
ELOVL5	Related to metabolism	Bt03235956_m1
ELOVL6	Related to metabolism	Bt00907566_m1
FADS2	Related to metabolism	Bt03256255_g1
FASN	Related to metabolism	Bt03210471_g1
AQP3	Related to metabolism	Bt03253663_m1
PGK1	Related to metabolism	Bt03225854_mH
SLC2A3	Related to metabolism	Bt03259513_g1
SLC2A5	Related to metabolism	Bt03258299_g1
AKR1B1	Related to metabolism	Bt03218049_g1
G6PD	Related to metabolism	Bt03649181_m1
AGPAT9	Related to metabolism	Bt04292093_m1
PPARA	Related to metabolism	Bt03220821_m1
PLIN2	Related to metabolism	Bt03212182_m1
PLIN3	Related to metabolism	Bt03230537_m1

* Probes are inventoried by the ThermoFischer Scientific company (no primer sequence information)
