# Supplementary material for: Tauroursodeoxycholic Acid Supplementation in In Vitro Culture of Indicine Bovine Embryos: Molecular and Cellular Effects on the In Vitro Cryotolerance
Source: Int J Mol Sci. 2023 Sep 14;24(18):14060. doi: 10.3390/ijms241814060 (PMC10531190; doi:10.3390/ijms241814060)
Supplement: Supplementary file 1 [file ijms-24-14060-s001.zip › Supplementary data Table S2.pdf]

Table S2: Fold Change values of all tested genes: hatched blastocysts collected on days 8-9. Control = 0 μM of TUDCA; T50 = 50 μM of TUDCA; T200 = 200 μM of TUDCA; T1000 = 1000 μM of TUDCA.

Gene Symbol	Control	Control	Control	Control	Control	T1000	T1000	T1000	T1000	T200	T200	T200	T200	T200	T50	T50	T50	T50	T50
ACACA	0.072742	0.083371	0.155465	0.081119	0.082057	0.162898	0.135538	0.145134	0.14744	0.069807	0.115177	0.112634	0.079535	0.103981	0.12435	0.068139	0.152845	0.107841	0.029759
ACSL3	1.345402	0.838713	0.770397	1.634997	1.548042	1.43584	1.682351	1.941946	1.67412	0.542416	1.686303	1.149759	1.120485	1.712373	1.661783	0.412051	0.756406	1.451679	0.382881
AQP3	1.087433	0.513697	1.080046	0.824716	1.167666	0.640866	0.451305	1.277856	0.717695	0.510385	0.586043	0.404398	0.418097	0.764826	0.873986	0.269521	0.68013	0.516972	0.266671
ACTB	18.72423	13.52913	4.832587	17.04441	18.34812	21.9464	19.23017	35.57227	24.67095	9.373377	13.77371	15.37125	11.71502	17.805	20.87522	12.54744	9.545597	17.99513	13.2758
AGPAT9	0.114234	0.123206	0.087602	0.095298	0.092499	0.144821	0.119991	0.144421	0.135905	0.133394	0.167101	0.117941	0.099498	0.138932	0.151631	0.11696	0.136397	0.154101	0.049054
AKR1B1	3.035926	2.65691	7.654777	3.246321	4.406611	2.090495	2.837238	1.282122	1.966639	2.823166	3.69359	2.433477	2.388887	4.524879	3.257076	1.89253	2.753952	3.584695	1.916365
ARO	0.007262	0.002328	0.007349	0.003101	0.001518	1.6E-296	0.01348	0.012006	1.4E-100	0.003711	0.003664	0.000566	0.009486	0.006426	0.004909	0.004226	0.002496	0.005539	0.003433
ATF4	0.08644	0.038699	0.009803	0.074947	0.058176	0.065492	0.084144	0.391973	0.129278	0.021221	0.063907	0.041872	0.042658	0.060744	0.05732	0.049468	0.038433	0.047458	0.103568
ATF6	0.07789	0.07666	0.125571	0.100169	0.177649	0.157059	0.153328	0.288016	0.190721	0.125495	0.052942	0.111959	0.08636	0.110912	0.127584	0.048975	0.136936	0.171044	0.037347
B2M	0.929597	0.865396	0.827426	1.043929	0.758572	0.839692	0.946619	0.830133	0.870658	1.091568	1.006348	0.883491	1.99335	1.307499	1.062378	1.179171	0.918879	1.085296	1.147644
BAX	0.607915	0.535543	0.625628	0.528377	0.97943	0.605232	0.940859	0.870706	0.791539	0.861172	0.602463	0.917289	0.673834	0.835697	0.967684	0.325319	0.599967	0.844284	0.764306
BID	0.03106	0.03822	0.017954	0.027927	0.031052	0.029342	0.029624	0.04048	0.032771	0.021087	0.049396	0.039068	0.019234	0.035382	0.05288	0.02936	0.025572	0.031988	0.040966
CASP3	0.001515	0.00298	4.1E-296	0.005071	0.002926	0.009838	0.002764	0.001555	0.003484	0.000785	0.001825	0.003751	0.002651	0.004241	0.003883	0.00366	0.002166	0.003864	0.000698
CASP9	0.055184	0.053935	0.038471	0.055676	0.057919	0.063929	0.092692	0.080778	0.078231	0.027373	0.109396	0.080602	0.076227	0.081348	0.096774	0.050184	0.040324	0.093747	0.038417
CAT	0.237807	0.161723	0.169361	0.393843	0.168667	0.190203	0.253123	0.235986	0.224826	0.161639	0.181516	0.219518	0.104117	0.232992	0.285382	0.07855	0.163469	0.205408	0.124984
CD40	0.010417	0.00947	0.028074	0.010509	0.004949	0.015242	0.007522	0.01919	0.013007	0.010928	0.023122	0.014292	0.015161	0.014714	0.01221	0.010762	0.013602	0.020878	0.002914
DDIT3	0.038748	0.044014	0.025618	0.02315	0.026882	0.039238	0.019151	0.08767	0.040391	0.067445	0.048105	0.042243	0.030997	0.017483	0.03846	0.016441	0.063957	0.033658	0.028488
DNMT1	0.06447	0.061352	0.035142	0.037131	0.025929	0.087525	0.053868	0.061971	0.066361	0.03979	0.068834	0.06673	0.027466	0.051064	0.041831	0.036886	0.065522	0.042034	0.048548
DNMT3A	2.676528	2.481485	3.487315	2.995279	3.7053	3.378162	4.549091	3.051969	3.606575	2.901914	3.908138	3.516121	2.720606	2.912028	3.815935	1.512936	2.72203	2.653082	1.597074
DNMT3B	0.834225	0.924526	0.897004	1.016408	0.731914	1.199599	0.867201	0.839519	0.955936	0.63556	1.387209	1.843862	0.918754	0.841985	1.627069	0.685525	1.604392	0.711647	0.431696
EIF2A	0.342884	0.585013	0.636484	0.325535	0.575331	0.678342	0.686409	0.665283	0.676673	0.548558	0.621743	0.513799	0.417438	0.522983	0.481495	0.453503	0.468626	0.496569	0.318744
ELOVL5	0.08952	0.128698	0.09951	0.129111	0.091192	0.238076	0.170034	0.18906	0.197083	0.137022	0.127546	0.089365	0.125404	0.14434	0.196187	0.093811	0.146669	0.120579	0.034835
ELOVL6	0.828203	0.66892	0.902789	0.648977	0.826778	1.130936	1.578338	0.507665	0.967769	0.704986	1.025389	1.14581	0.731476	1.203294	0.933362	1.338513	1.242948	1.271758	0.169117
FADS2	0.670478	0.3326	1.045767	0.744605	1.091055	0.830588	0.871536	0.58458	0.750824	0.477923	1.285638	0.917348	0.718529	0.582581	1.130908	0.305594	0.72782	1.056848	0.113931
FASN	0.397602	0.262872	0.303278	0.456123	0.348811	0.537927	0.341033	0.510064	0.454026	0.342196	0.531032	0.258465	0.35295	0.490579	0.58316	0.250206	0.317208	0.474469	0.190271
FOXO3	0.012383	0.013296	0.016045	0.012442	0.012607	0.028706	0.018352	0.046467	0.029038	0.013858	0.02354	0.013974	0.014878	0.017549	0.029711	0.009942	0.022647	0.018737	0.013263
G6PD	0.297343	0.413179	0.331812	0.243933	0.239128	0.218484	0.318615	0.633111	0.353255	0.299761	0.335695	0.23014	0.226441	0.38914	0.29921	0.486961	0.247588	0.364405	0.148634
GAPDH	2.955072	2.088624	3.44308	3.38154	3.167661	2.083607	1.648111	1.891338	1.865908	3.124816	2.083237	2.424115	2.369644	3.573224	2.186229	3.069618	3.904223	2.786567	1.928257
GATM	0.012426	0.007798	0.014559	0.007034	0.005326	0.015294	0.011035	0.016284	0.014009	0.009261	0.030772	0.010971	0.007674	0.012065	0.015371	0.004987	0.006701	0.004336	0.006282
GFPT2	0.016046	0.020137	0.011305	0.004704	0.021198	0.020318	0.032331	0.028211	0.026465	0.014456	0.018413	0.009366	0.009794	0.006207	0.020289	0.007469	0.007333	0.016121	0.004605
GLRX2	0.389365	0.571528	0.535922	0.363007	0.403257	0.554805	0.602475	0.44326	0.529191	0.309586	0.626376	0.641987	0.424425	0.502715	0.401796	0.298285	0.505994	0.56152	0.48861
GPX1	0.166737	0.18912	0.246147	0.191829	0.142318	0.349481	0.193682	0.605701	0.344845	0.142064	0.202495	0.195989	0.179616	0.232934	0.319057	0.141882	0.199164	0.224672	0.186256
GPX4	0.136927	0.099262	0.052965	0.089489	0.074872	0.174935	0.124545	0.395907	0.2051	0.135031	0.123148	0.107946	0.070225	0.207203	0.103665	0.166654	0.089461	0.131905	
GSK3A	0.219461	0.233408	0.266702	0.149567	0.16768	0.242534	0.231553	0.287331	0.252717	0.15211	0.241957	0.136434	0.124944	0.194263	0.207368	0.154942	0.275724	0.126771	0.18499
HAND1	0.001074	0.005983	0.007424	0.006804	0.004574	0.02675	0.004304	0.04151	0.016845	1.5E-296	0.003894	0.00705	0.009601	0.008001	0.01269	0.002544	0.00944	0.002155	0.000826
HIF1A	0.015317	0.013522	0.014387	0.010196	0.008566	0.052619	0.022248	0.026961	0.031605	0.017874	0.030995	0.020291	0.008794	0.021429	0.016037	0.029994	0.022952	0.015392	0.008328
HMBS	0.095749	0.082415	0.178278	0.106495	0.100965	0.15055	0.13749	0.166444	0.151045	0.082426	0.11338	0.094487	0.078215	0.102304	0.14308	0.043317	0.103554	0.07804	0.072047
HMOX1	0.177536	0.08082	0.069024	0.111237	0.084634	0.252871	0.197303	0.216643	0.221119	0.129991	0.119341	0.043317	0.052562	0.081664	0.217647	0.057748	0.098204	0.114008	0.085609
HP1	0.037376	0.07423	0.054121	0.030437	0.02971	0.054681	0.060753	0.085209	0.065664	0.043826	0.085155	0.051198	0.043508	0.045376	0.089405	0.053221	0.083795	0.055763	0.055753
HPRT1	0.20333	0.293228	0.131288	0.215526	0.200236	0.244203	0.239773	0.236525	0.240165	0.164601	0.154597	0.286241	0.089892	0.138107	0.15174	0.142114	0.204771	0.172569	0.157766
HSPA1A	0.003346	0.003488	4.1E-296	0.003592	0.001156	0.003442	0.000897	0.003073	0.002117	0.002039	0.00334	0.000946	0.001521	0.005248	0.002689	0.002343	0.002188	0.001288	0.001406
HSPA5	3.222967	2.556902	3.305209	2.913848	2.716267	3.35091	2.971619	2.305826	2.842458	3.070609	3.001906	2.916384	2.592967	2.133627	3.039009	1.697808	3.502228	3.662986	0.453884
HSPD1	1.202466	2.256088	1.977881	0.897966	1.213327	1.923616	1.609519	2.267305	1.914879	1.53863	1.957951	1.404099	0.941728	1.247519	1.460257	1.119383	1.32612	0.87323	0.689524
IFITM3	0.228468	0.120046	0.15387	0.076481	0.154605	0.423728	0.159655	0.969944	0.403372	0.212566	0.120023	0.166921	0.123757	0.020164	0.298273	0.048217	0.142743	0.320683	0.160543
IGFBP2	0.228468	0.120046	0.15387	0.076481	0.154605	0.423728	0.159655	0.969944	0.403372	0.212566	0.120023	0.166921	0.123757	0.020164	0.298273	0.048217	0.142743	0.320683	0.160543
IGFBP4	0.007144	0.002911	4.1E-296	0.001789	0.002757	0.009592	0.00185	0.003911	0.00411	0.007016	0.0087	0.004997	0.003581	4.6E-297	0.006634	0.002542	0.003797	0.006516	0.009104
KEAP1	0.100662	0.10481	0.115643	0.108474	0.092756	0.168109	0.116956	0.200428	0.157964	0.177356	0.194081	0.115215	0.116432	0.173195	0.179581	0.048637	0.131034	0.156904	0.069202
KRT8	18.35789	15.00502	30.86724	17.91178	23.37012	25.15249	33.72709	27.25211	28.48961	23.13833	20.60374	19.99214	15.1199	24.32978	25.59689	11.55801	16.9427	21.72813	9.881656
LUM	0.001347	0.005887	0.001661	0.005995	0.003728	0.211691	0.022731	0.00435	0.02756	0.030033	0.035153	0.014165	5.2E-297	0.000986	0.000973	0.068507	0.060134	0.006332	0.046716
MTIF3	0.003723	0.002119	4.1E-296	0.004091	0.006398	0.004631	0.003455	0.014652	0.006166	0.005619	0.002104	0.00518	0.001455	0.006063	0.004585	0.00061	0.003694	0.000563	0.00549
NANOG	0.022645	0.05604	0.009819	0.048888	0.009954	0.017678	0.017368	0.044659	0.023937	0.020905	0.037437	0.096489	0.028101	4.6E-297	0.104355	0.013673	0.06503	0.028736	0.017153
NFE2L2	0.088774	0.221101	0.069412	0.035965	0.06701	0.185774	0.085933	0.204702	0.148407	0.159516	0.217751	0.185726	0.100014	0.108839	0.188435	0.057257	0.095507	0.071282	0.033593
NFkB2	0.190049	0.228726	0.135948	0.097852	0.107747	0.219784	0.201976	0.251136	0.223409	0.19178	0.229189	0.223094	0.273003	0.201181	0.231608	0.18299	0.29714	0.208867	0.181853
PAF1	0.085546	0.063313	0.053425	0.071553	0.059047	0.077514	0.064537	0.13671	0.088111	0.080289	0.067325	0.050855	0.042211	0.062445	0.070384	0.054178	0.073874	0.068852	0.006232
PGK1	0.095474	0.091021	0.072894	0.106987	0.080098	0.120304	0.09152	0.16061	0.120936	0.156044	0.105503	0.043873	0.079642	0.114551	0.132467	0.152159	0.094136	0.121538	0.070703
PLIN2	0.330435	0.220442	0.265562	0.143889	0.227572	0.177877	0.173901	0.267557	0.202292	0.272872	0.314097	0.408543	0.176028	0.16324	0.35957	0.168654	0.223719	0.256095	0.172601
PLIN3	3.579641	2.444258	3.550093	2.957878	5.305608	3.045667	2.934205	2.841856	2.939622	2.087084	4.1627	3.417896	3.439099	4.178101	3.282421	1.031111	3.181636	4.585071	1.498036
POU5F1	0.928682	0.801654	1.499818	0.944298	0.87157	0.773735	0.809098	2.63288	1.181339	0.662137	0.876497	1.132844	1.185525	0.572292	2.172698	0.345445	1.096859	1.252459	1.425201
PPARA	0.001867	0.002414	4.1E-296	0.003102	0.005049	0.015844	0.003728	0.014279	0.009449	0.029196	0.019223	0.002908	0.003622	0.006909	0.004465	0.004159	5E-297	0.002337	0.013443
PPIA	4.215599	3.426427	6.703949	3.607915	5.096966	4.085513	3.714948	4.266548	4.015983	4.389566	4.862941	3.419145	3.862274	4.159403	4.681414	4.505266	4.241578	4.188068	4.337467
PRDX1	6.329426	6.065622	6.676868	5.197941	6.879713	5.757817	5.950433	10.1591	7.034833	7.895707	8.375429	4.327557	7.053516	7.70927	8.866955	8.354362	4.783223	5.626828	5.811587
PRDX3	0.057553	0.109441	0.059857	0.06485	0.062298	0.137661	0.131191	0.19396	0.151883	0.134228	0.168964	0.084731	0.056881	0.133764	0.123658	0.056797	0.10035	0.069652	0.067085
REST	0.107072	0.139018	0.071365	0.144021	0.076813	0.239453	0.136043	0.41742	0.238702	0.098892	0.16739	0.171899	0.109583	0.16103	0.195334	0.059188	0.108609	0.081462	0.075239
RPL15	0.011771	0.015467	0.003206	0.01158	0.012259	0.027029	0.007296	0.006952	0.01111	0.004101	0.003436	0.012842	0.00776	0.012289	0.011181	0.014143	0.012951	0.004685	0.016182
RPL30	1.976576	2.294892	3.563357	1.59503	1.619962	2.359274	2.871935	2.921821	2.705425	3.513898	2.832499	3.318378	1.94081	2.097469	2.079124	2.667551	3.17034	1.766351	2.175109
S100A10	1.309945	1.455242	0.769634	0.978196	1.135705	1.448351	1.222916	1.456396	1.371566	0.842408	1.11514	1.028213	0.6615	1.151349	1.395189	1.258973	1.174184	1.051387	1.00316
S100A14	2.334973	1.152774	2.069674	3.652217	3.194229	1.997778	1.809332	2.980497	2.208774	1.608783	1.972063	1.560058	1.89557	2.851653	2.998004	0.553804	1.117897	2.950103	2.319554
SLC2A3	0.756588	0.974346	0.843259	1.037859	0.922442	0.817085	0.93941	1.000272	0.915757	0.868779	1.211037	0.81641	0.816843	0.870209	1.356946	0.549166	0.756802	1.193853	0.166746
SLC2A5	0.320322	0.230889	0.075771	0.734248	0.428173	0.599803	0.290163	0.811835	0.520882	0.089677	0.309432	0.254343	0.194728	0.491102	0.344097	0.112667	0.140419	0.424525	0.146673
SOD1	0.476999	0.79824	0.809473	0.58524	0.505757	0.809402	0.583457	0.603218	0.658035	0.635862	1.0056	0.716799	0.518773	1.03185	0.529702	0.429576	0.586445	0.821042	0.385176
SOX2	0.003628	0.001816	0.005561	0.002108	0.003534	0.015648	0.002879	0.031368	0.011223	0.001334	0.005377	0.006167	0.004206	4.6E-297	0.013643	0.001495	0.004736	0.006476	0.006132
SREBF1	0.026399	0.034046	0.052172	0.053684	0.047966	0.087689	0.038689	0.038333	0.050668	0.036963	0.036045	0.023581	0.016672	0.079032	0.027619	0.026129	0.026234	0.037942	0.010894
SREBF2	0.157828	0.137516	0.126492	0.179689	0.205356	0.422889	0.420148	0.324205	0.386233	0.172034	0.328308	0.215603	0.178446	0.313572	0.25743	0.13607	0.194005	0.261364	0.091145
TXNRD1	0.195586	0.224203	0.266938	0.144132	0.238564	0.295249	0.440922	0.557058	0.417046	0.294391	0.26536	0.251634	0.190471	0.339219	0.369341	0.198733	0.223745	0.385971	0.181564
XBP1	0.411361	0.31914	0.530501	0.394471	0.395627	0.555215	0.531484	0.412579	0.495662	0.405598	0.473709	0.478155	0.060887	0.37155	0.459414	0.166878	0.423484	0.416966	0.199181
