# Supplementary material for: Tauroursodeoxycholic Acid Supplementation in In Vitro Culture of Indicine Bovine Embryos: Molecular and Cellular Effects on the In Vitro Cryotolerance
Source: Int J Mol Sci. 2023 Sep 14;24(18):14060. doi: 10.3390/ijms241814060 (PMC10531190; doi:10.3390/ijms241814060)
Supplement: Supplementary file 1 [file ijms-24-14060-s001.zip › Supplementary data Table S3.pdf]

Table S3: Fold Change values of all tested genes: hatched embryos collected 24- and 48-hours post-warming. Control = 0 μM of TUDCA; T200 = 200 μM of TUDCA.

Gene Symbol	Control	Control	Control	Control	T200	T200	T200
ACACA	0.020964	0.018653	0.036348	0.035991	0.017647	0.020053	0.02328
ACSL3	0.499423	0.284436	0.46955	0.649971	0.189058	0.935528	1.311303
AQP3	0.258977	0.301775	0.241916	0.306079	0.256937	0.255644	0.624477
ACTB	5.065591	4.680116	6.661714	8.500149	4.616212	6.102507	8.145309
AGPAT9	0.035315	0.060356	0.050949	0.03249	0.031629	0.032196	0.040697
AKR1B1	1.640154	1.431481	1.171836	0.976882	1.113985	0.962934	0.804854
ARO	0.002126	0.003318	0.003242	0.002857	0.001548	0.002253	0.019287
ATF4	0.012666	0.008867	0.016154	0.023435	0.008827	0.005355	0.013908
ATF6	0.008781	0.013419	0.060381	0.03831	0.03146	0.042761	0.117026
B2M	0.389521	0.588998	0.45591	0.223711	0.508055	0.569331	0.93011
BAX	0.210759	0.185051	0.271169	0.278935	0.146918	0.117339	0.240494
BID	0.010269	0.01728	0.016134	0.006609	0.015025	0.028425	0.032473
CASP3	0.001048	0.001075	0.001493	0.001426	0.000744	0.000995	0.004668
CASP9	0.029834	0.030806	0.034873	0.025416	0.023321	0.031989	0.102554
CAT	0.083635	0.052127	0.079413	0.075599	0.076957	0.131755	0.170389
CD40	0.003259	0.003515	0.008793	0.005024	0.002733	0.003991	0.006071
DDIT3	0.007335	0.007743	0.021897	0.006402	0.007013	0.011161	0.009148
DNMT1	0.017661	0.017862	0.012945	0.01532	0.008965	0.016818	0.045773
DNMT3A	1.125329	0.884875	1.124542	1.009384	0.860231	0.552479	0.553112
DNMT3B	0.309759	0.317314	0.238654	0.349609	0.221845	0.127586	0.172924
EIF2A	0.171029	0.243689	0.200734	0.100404	0.130537	0.287838	0.32671
ELOVL5	0.050933	0.076467	0.051205	0.037188	0.065773	0.135569	0.201263
ELOVL6	0.292133	0.335271	0.197707	0.156095	0.19138	0.480872	0.496256
FADS2	0.298907	0.202367	0.244185	0.292517	0.206896	1.539482	1.277135
FASN	0.141076	0.189455	0.123902	0.153812	0.156935	0.056067	0.108444
FOXO3	0.009203	0.003861	0.008156	0.005031	0.006008	0.008021	0.028968
G6PD	0.09677	0.179832	0.12597	0.085677	0.159571	0.250875	0.542983
GAPDH	1.2329	0.949693	0.440886	0.85089	0.905616	3.189198	1.515432
GATM	0.003838	0.001566	0.009587	0.008305	0.001851	0.004517	0.010615
GFPT2	0.006851	0.007378	0.005188	0.004875	0.00669	0.009594	0.007235
GLRX2	0.130825	0.164071	0.127498	0.091484	0.139386	0.037702	0.04058
GPX1	0.083458	0.075739	0.091066	0.09588	0.094564	0.131717	0.186901
GPX4	0.042286	0.050497	0.029869	0.059126	0.036627	0.068632	0.069695
GSK3A	0.05415	0.068649	0.071528	0.070038	0.064544	0.075953	0.166654
HAND1	0.000968	0.000674	0.00064	0.001129	0.001128	0.003406	0.031318
HIF1A	0.004308	0.008655	0.009628	0.004658	0.003758	0.01547	0.038432
HMBS	0.040377	0.030564	0.039014	0.035437	0.02569	0.015004	0.011186
HMOX1	0.09794	0.055611	0.055808	0.07034	0.05281	0.036002	0.03577
HP1	0.016613	0.02266	0.024078	0.012517	0.01649	0.00551	0.006488
HPRT1	0.067279	0.079805	0.069171	0.055392	0.087595	0.060425	0.058463
HSPA1A	0.000325	0.000744	0.00072	0.000913	0.000222	0.000571	0.001372
HSPA5	0.771705	0.859802	1.452762	0.800788	0.771143	0.565498	0.675059
HSPD1	0.45088	0.540375	0.517006	0.29092	0.583689	0.616332	0.823476
IFITM3	0.001441	0.006728	0.010073	0.001566	0.013712	0.001465	0.019184
IGFBP2	0.013913	0.034751	0.049919	0.054317	0.058593	0.008365	0.042058
IGFBP4	0.000762	0.001879	0.001632	0.000655	0.001314	0.000157	0.030138
KEAP1	0.040522	0.038717	0.064676	0.048564	0.042952	0.04339	0.093919
KRT8	6.543332	5.731473	8.764871	6.524161	4.247452	4.264197	5.495975
MTIF3	0.001629	0.001468	0.003519	0.000859	0.001286	0.002824	0.000686
NANOG	0.001183	0.009544	0.004275	0.010359	0.00877	0.003432	0.016332
NFE2L2	0.028642	0.035197	0.053603	0.037566	0.044101	0.111404	0.127907
NFkB2	0.030612	0.059775	0.084016	0.052995	0.053209	0.011629	0.010962
PAF1	0.020557	0.036234	0.031426	0.025805	0.020118	0.071747	0.171342
PGK1	0.052851	0.080907	0.038979	0.030728	0.057167	0.066785	0.051376
PLIN2	0.079941	0.092854	0.107292	0.074473	0.084561	0.138582	0.282484
PLIN3	1.635485	1.072847	1.129048	1.473752	0.881547	0.229774	0.299923
POU5F1	0.213381	0.222001	0.355504	0.279845	0.300016	0.218193	1.001654
PPIA	1.715144	1.683531	1.246991	1.069587	1.607036	1.567653	1.260317
PRDX1	2.372117	2.783276	1.656027	1.55351	2.362601	7.118065	5.101633
PRDX3	0.042314	0.036453	0.024147	0.030702	0.028713	0.032793	0.086296
REST	0.058014	0.05584	0.078911	0.08349	0.052582	0.173663	0.283958
RPL15	0.003735	0.002513	0.008141	0.009474	0.000166	0.013556	0.005646
RPL30	0.66607	0.800835	0.9179	0.665766	0.641296	0.321495	0.386397
S100A10	0.342959	0.51472	0.275693	0.330619	0.30648	0.895554	1.383877
S100A14	1.105156	0.6022	0.700057	1.138273	0.37755	0.625653	0.350826
SLC2A3	0.354705	0.371351	0.217363	0.280181	0.328747	0.623366	0.728985
SLC2A5	0.073844	0.066828	0.14206	0.232735	0.030024	0.009438	0.030621
SOD1	0.283942	0.244129	0.320243	0.190976	0.208252	0.188571	0.249459
SOX2	0.000572	0.001289	0.001697	0.001247	0.000604	0.000709	0.014983
SREBF1	0.01579	0.008851	0.020996	0.009284	0.005745	0.01468	0.045848
SREBF2	0.051298	0.042415	0.043896	0.066513	0.037422	0.004723	0.016717
TXNRD1	0.046089	0.063896	0.103432	0.064911	0.068523	0.059271	0.126892
XBP1	0.109959	0.131812	0.165811	0.252728	0.024436	0.063385	0.091948
